# Copper Modulated Lead‐Free Cs_4_MnSb_2_Cl_12_ Double Perovskite Microcrystals for Photocatalytic Reduction of CO_2_

**DOI:** 10.1002/advs.202307543

**Published:** 2023-12-09

**Authors:** Bo Gao, Changqing Tian, Linfeng Guo, Jinchen Zhou, Zixian Wang, Chengfan Fu, Hongmei Ran, Wei Chen, Qiang Huang, Daofu Wu, Xiaosheng Tang, Zhongtao Luo

**Affiliations:** ^1^ School of Materials Science and Engineering Zhengzhou University Zhengzhou 450001 China; ^2^ College of Optoelectronic Engineering Chongqing University of Posts and Telecommunications Chongqing 400065 China; ^3^ State Key Laboratory of Catalysis Dalian Institute of Chemical Physics Chinese Academy of Sciences Dalian 116023 China; ^4^ Key Laboratory of Optoelectronic Technology & Systems (Ministry of Education) College of Optoelectronic Engineering Chongqing University Chongqing 400044 China

**Keywords:** catalytic mechanism, Cu‐doped Cs_4_MnSb_2_Cl_12_, lead‐free perovskite microcrystals, photocatalytic CO_2_ reduction

## Abstract

In order to deal with the global energy crisis and environmental problems, reducing carbon dioxide through artificial photosynthesis has become a hot topic. Lead halide perovskite is attracted people's attention because of its excellent photoelectric properties, but the toxicity and long‐term instability prompt people to search for new photocatalysts. Herein, a series of <111> inorganic double perovskites Cs_4_Mn_1‐x_Cu_x_Sb_2_Cl_12_ microcrystals (x = 0, 0.1, 0.2, 0.3, 0.4, and 0.5) are synthesized and characterized. Among them, Cs_4_Mn_0.7_Cu_0.3_Sb_2_Cl_12_ microcrystals have the best photocatalytic performance, and the yields of CO and CH_4_ are 503.86 and 68.35 µmol g^−1^, respectively, after 3 h irradiation, which are the highest among pure phase perovskites reported so far. In addition, in situ Fourier transform infrared (FT‐IR) spectroscopy and electron spin resonance (ESR) spectroscopy are used to explore the mechanism of the photocatalytic reaction. The results highlight the potential of this class of materials for photocatalytic reduction reactions.

## Graphical abstract

In this paper, a series of <111> inorganic double perovskite Cs_4_Mn_1‐x_Cu_x_Sb_2_Cl_12_ microcrystals are synthesized, and their photocatalytic efficiency is improved by introducing Cu element, and their photoelectric properties are significantly enhanced. After various stability tests, the photocatalyst maintained good stability. Furthermore, in situ FT‐IR spectroscopy and ESR spectroscopy are adopted to precisely unraveled active sites, reaction intermediates, and photocatalytic reduction process.

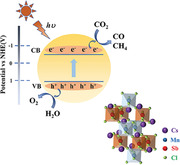

## 1 Introduction

In recent years, the continuous increase in industrialization and the excessive consumption of fossil fuels have triggered an energy crisis on a global scale, the large amount of CO_2_ emitted has caused many environmental problems.^[^ ^1^, ^2^, ^3^, ^4^ ^]^ As a result, people have developed a strong interest in obtaining resources from nature. Among them, the solar energy, as a clean, cheap, sustainable energy, has great potential.^[^ ^5^ ^]^ Photocatalytic reduction of CO_2_ through artificial photosynthesis can not only reduce the content of CO_2_ in the atmosphere, but also produce CO, methane and other chemical fuels.^[^ ^6^ ^]^ In the past few years, many semiconductor photocatalysts have been investigated for application in this artificial photosynthesis process, but the conversion efficiency is still low.^[^ ^7^, ^8^ ^]^ Lead halide perovskites have attracted much attention in solar cells, light emitting diodes, and photodetectors due to their excellent photoelectric properties,^[^ ^9^, ^10^, ^11^ ^]^ such as large absorption coefficient, adjustable band gap, excellent visible light capture ability, and high charge carrier mobility.^[^ ^12^, ^13^, ^14^ ^]^ However, the toxicity of lead and long‐term instability greatly limit its development potential, and lead‐free perovskite will be the next research direction.^[^ ^15^, ^16^ ^]^

Recently, an inorganic double perovskite of type <111> has been reported.^[^ ^17^, ^18^ ^]^ Compared with traditional double perovskites, These perovskites use metallic elements of different oxidation states, such as Sb^3+^ and Bi^3+^, to replace Pb^2+^ and generate ordered vacancy in the whole lattice to compensate for the charge difference, thus forming layered perovskite structure.^[^ ^19^ ^]^ The thickness of the inorganic sheets can be increased with a heterovalent replacement of the trivalent metal by two metals, M′^II^ and M^III^, yielding the triple‐layer structure A_4_M′^II^M^III^_2_X_12_ (M = Bi^3+^ or Sb^3+^; M′ = Cd^2+^, Mn^2+^, and or Cu^2+^).^[^ ^20^ ^]^ Metallic elements such as antimony and bismuth replace lead, improving overall stability while reducing toxicity.^[^ ^21^, ^22^ ^]^ At the same time, they have been shown to have a suitable bandgap for photovoltaic power generation, as well as tunable optical and magnetic properties, which have attracted people's interest in many fields.^[^ ^17^, ^20^, ^23^ ^]^ Qi and his group successfully synthesized Cs_4_MnBi_2_Cl_12_ single crystals using hydrothermal methods and applied them to photocatalytic degradation of organic pollutants.^[^ ^24^ ^]^ Mai and his team used the colloidal method to synthesize Cs_4_ZnSb_2_Cl_12_ nanoparticles and adjust their size to to enhance the visible‐light photocatalytic performance for toluene oxidation.^[^ ^25^ ^]^ Cai and his group report the first colloidal synthesis of Cs_4_CuSb_2_Cl_12_ perovskite‐type nanocrystals (NCs) as high‐speed photodetectors with ultrafast photoresponse and narrow bandwidth.^[^ ^26^ ^]^ In our previous work, we synthesized Cs_4_CuSb_2_Cl_12_ quantum dots by hot injection approach and applied them to photocatalytic CO_2_ reduction, with a CO yield of 233 µmol g^−1^ and a CH_4_ yield of 74 µmol g^−1^ after irradiation for 3 h.^[^ ^27^ ^]^

Based on our previous work, a series of <111> inorganic double perovskite Cs_4_Mn_1‐x_Cu_x_Sb_2_Cl_12_ microcrystals (x = 0, 0.1, 0.2, 0.3, 0.4, and 0.5) were synthesized and characterized in this work. The synthesized Cs_4_Mn_1‐x_Cu_x_Sb_2_Cl_12_ microcrystals can still maintain good stability after heating at 100 °C or irradiation at 365 nm UV lamp for 100 h. By systematically changing the ratio of copper to manganese, it improves its carbon dioxide reduction efficiency. The electron consumption employed to generate CO and CH_4_ was, respectively, 1007.72, and 546.80 µmol g^−1^ over the Cs_4_Mn_0.7_Cu_0.3_Sb_2_Cl_12_ microcrystals under 3 h of illumination, which is the highest among pure phase perovskites reported so far. In addition, in situ Fourier transform infrared (FT‐IR) spectroscopy and electron spin resonance (ESR) spectroscopy are used to explore the mechanism of the photocatalytic reaction. Our results highlight the potential of this class of materials for photocatalytic reduction reactions.

## 2 Results and Discussion

### 2.1 Crystal Structure and Morphology of Cs_4_Mn_1‐x_Cu_x_Sb_2_Cl_12_ Microcrystals

**Figure 1:**
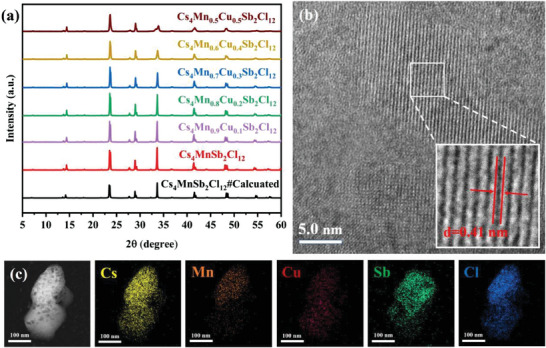
a) XRD patterns of Cs_4_Mn_1‐x_Cu_x_Sb_2_Cl_12_ microcrystals. b) HRTEM images of the Cs_4_Mn_0.7_Cu_0.3_Sb_2_Cl_12_ microcrystals. c) EDX mapping images of the Cs_4_Mn_0.7_Cu_0.3_Sb_2_Cl_12_ microcrystals.

In order to verify the phase purity of Cs_4_Mn_1‐x_Cu_x_Sb_2_Cl_12_ (x = 0, 0.1, 0.2, 0.3, 0.4, and 0.5) microcrystals, X‐ray diffraction was performed. The diffraction peaks of the obtained Cs_4_Mn_1‐x_Cu_x_Sb_2_Cl_12_ microcrystals are shown in **Figure** **1** (a), with the addition of Cu element, the position of diffraction peak does not move, which are completely consistent with those reported previously.^[^ ^20^ ^]^ In addition, no additional diffraction peaks are found, indicating high purity of the synthesized Cs_4_Mn_1‐x_Cu_x_Sb_2_Cl_12_ microcrystals. In order to better understand the morphology of microcrystals, the measurements were made by scanning electron microscope (SEM) and transmission electron microscope (TEM). Figure S1 (Supporting Information) (a–f) shows the SEM image of Cs_4_Mn_1‐x_Cu_x_Sb_2_Cl_12_, and it can be seen that the material has a microcrystalline structure. High‐resolution TEM (HRTEM) images of Cs_4_Mn_0.7_Cu_0.3_Sb_2_Cl_12_ microcrystals (Figure 1 (b)) show high crystallinity with a lattice spacing of 0.41 nm. This smaller grain size has a larger surface area, thus improving the adsorption of the reactants. Smaller particle sizes have a larger surface area and can provide more active sites for carbon dioxide adsorption and photocatalytic conversion.^[^ ^29^, ^30^ ^]^ The EDX mapping proves the presence and uniform distribution of Cs, Mn, Cu, Sb, and Cl in Cs_4_Mn_0.7_Cu_0.3_Sb_2_Cl_12_ microcrystals (Figure 1 (c)). The TEM and HRTEM images and EDX mapping results of Cs_4_MnSb_2_Cl_12_ microcrystals are shown in Figure S2 (Supporting Information), and the lattice spacing is 0.43 nm. In addition, we used Inductively Coupled Plasma‐Atomic Emission Spectrometry (ICP‐AES) to quantitatively analyze the elements of Cs_4_Mn_1‐x_Cu_x_Sb_2_Cl_12_ microcrystals. Table S2 (Supporting Information) shows the elemental contents of Mn and Cu, which is consistent with the stoichiometric ratio, further proving that we have successfully synthesized Cs_4_Mn_1‐x_Cu_x_Sb_2_Cl_12_ microcrystals.

### 2.2 Chemical Composition of the Cs_4_Mn_1‐x_Cu_x_Sb_2_Cl_12_ Microcrystals

**Figure 2:**
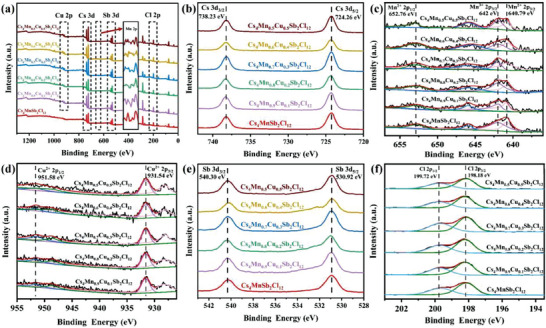
a) XPS spectra, b) Cs 3d, c) Mn 2p, d) Cu 2p, e) Sb 3d, f) Cl 2p of the Cs_4_Mn_1‐x_Cu_x_Sb_2_Cl_12_ microcrystals.

The surface chemical properties of Cs_4_Mn_1‐x_Cu_x_Sb_2_Cl_12_ microcrystals were further studied by X‐ray spectrum (**Figure** **2** (a)). As can be seen from Figure 2 (c), the binding energies of Mn 2p_3/2_ and Mn 2p_1/2_ are 640.79 and 652.76 eV respectively, corresponding to Mn^2+^. The binding energy peak at 642 eV is the 2p_3/2_ hybrid of Mn^3+^. The satellite peak at 645.8 eV may be the oxidation state of Mn^2+^. The coexistence of polyvalent states of elements can promote the photocatalytic performance.^[^ ^31^, ^32^, ^33^ ^]^ A similar situation also appears in the XPS spectrum of Cu (Figure 2 (d)), the two peaks with binding energy of 931.54 and 951.58 eV belong to Cu^2+^ 2p_3/2_ and Cu^2+^ 2p_1/2_, respectively, while the two satellite peaks at 928.13 and 948.61 eV belong to Cu^+^ 2p_3/2_ and Cu^+^ 2p_1/2_, respectively.^[^ ^34^, ^35^, ^36^ ^]^ The XPS spectral peaks of Cs, Sb, and Cl elements are shown in Figure 2 (b, e, and f). The XPS peaks of all the above elements did not shift, which further indicated that high purity Cs_4_Mn_1‐x_Cu_x_Sb_2_Cl_12_ microcrystals were synthesized.

### 2.3 The Stability of the Cs_4_Mn_1‐x_Cu_x_Sb_2_Cl_12_ Microcrystals

Stability is an important criterion for double perovskite photocatalyst to meet practical application. First, we conducted TGA test on Cs_4_Mn_1‐x_Cu_x_Sb_2_Cl_12_ microcrystals. As shown in Figure S3 (Supporting Information), Cs_4_Mn_1‐x_Cu_x_Sb_2_Cl_12_ microcrystals began to decompose at ≈300 °C and decomposed for a second time at ≈600 °C, indicating that Cs_4_Mn_1‐x_Cu_x_Sb_2_Cl_12_ microcrystals have good thermal stability. In addition, the stability of Cs_4_Mn_1‐x_Cu_x_Sb_2_Cl_12_ were evaluated by XRD, SEM, and XPS after high temperature treatment at 100 °C and 365 nm UV lamp irradiation. The XRD test results are shown in Figure S4 (Supporting Information) (a≈f). The XRD patterns of the Cs_4_Mn_1‐x_Cu_x_Sb_2_Cl_12_ microcrystals treated by the two methods is consistent with the original XRD patterns. This indicates that no obvious phase transition occurs in the Cs_4_Mn_1‐x_Cu_x_Sb_2_Cl_12_ microcrystals after the treatment with the two methods. In addition, the SEM images of the Cs_4_Mn_1‐x_Cu_x_Sb_2_Cl_12_ microcrystals treated in the two ways are shown in Figures S5 (a≈f) and S6 (a≈f) (Supporting Information), and the XPS spectra are shown in Figures S7–S12 (Supporting Information), which are basically consistent with the untreated Cs_4_Mn_1‐x_Cu_x_Sb_2_Cl_12_ microcrystals. The above results show that Cs_4_Mn_1‐x_Cu_x_Sb_2_Cl_12_ microcrystals have good thermal and optical stability. This makes it more possible for Cs_4_Mn_1‐x_Cu_x_Sb_2_Cl_12_ microcrystals to meet practical applications.

### 2.4 Optical Properties of the Cs_4_Mn_1‐x_Cu_x_Sb_2_Cl_12_ Microcrystals

The bandgap plays an important role in the photocatalytic reaction. A smaller band gap can make the photocatalysts possess higher visible light capture. As shown in Figure S13 (Supporting Information) (a), the optical properties of Cs_4_Mn_1‐x_Cu_x_Sb_2_Cl_12_ microcrystals were studied by UV–vis absorption spectra. With the introduction of Cu, the initial absorption band of Cs_4_Mn_1‐x_Cu_x_Sb_2_Cl_12_ microcrystals can be adjusted from ≈450 to ≈1500 nm wavelength, and better visible light response can be obtained. (αhν)^2^ versus hν curves were used to analyze the band gap of Cs_4_Mn_1‐x_Cu_x_Sb_2_Cl_12_ microcrystals, as shown in Figure S13 (b) (Supporting Information). All materials show direct bandgaps, which is consistent with the work previously reported.^[^ ^20^ ^]^ The band gap decreases with the addition of Cu element. Even at x = 0.1, the bandgap is sharply reduced from 2.98 to 1.68 eV, which indicates that even a small Cu element content will have an impact on the electronic structure of the material. This is consistent with previously reported studies.^[^ ^37^ ^]^

### 2.5 The Energy Band Structures of the Cs_4_Mn_1‐x_Cu_x_Sb_2_Cl_12_ Microcrystals

Suitable band structure is the key to obtain efficient photocatalytic reduction ability. The conduction band (CB) at the higher energy level can obtain photogenerated electrons with stronger reducing ability, which is more favorable for the photocatalytic reduction reaction.^[^ ^38^ ^]^ In order to study the band structure of Cs_4_Mn_1‐x_Cu_x_Sb_2_Cl_12_ microcrystals, valence band XPS (VB XPS) was used to obtain the valence band positions of Cs_4_Mn_1‐x_Cu_x_Sb_2_Cl_12_ microcrystals. As shown in Figure S14 (a–f) (Supporting Information), the VB of Cs_4_Mn_1‐x_Cu_x_Sb_2_Cl_12_ microcrystals are 1.39, 1.05, 1.04, 1.05, 1.01, and 1.00 eV, respectively. In addition, we can use the equation: E_CB_ = E_VB –_ Eg to determine the corresponding conduction band (CB) positions. The calculated band structure is shown in Figure S15 (Supporting Information), Cs_4_Mn_1‐x_Cu_x_Sb_2_Cl_12_ microcrystals all have appropriate bandgaps for photocatalytic reduction. Therefore, we further studied the photocatalytic reduction ability of Cs_4_Mn_1‐x_Cu_x_Sb_2_Cl_12_ microcrystals for carbon dioxide.

### 2.6 Photocatalytic Activity

**Figure 3:**
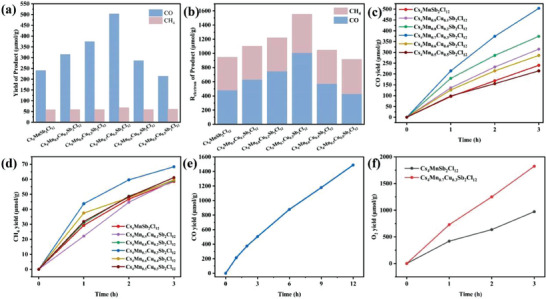
a) Comparison of photocatalytic carbon dioxide reduction properties of Cs_4_Mn_1‐x_Cu_x_Sb_2_Cl_12_ microcrystals. b) *R*_electron_ over the Cs_4_Mn_1‐x_Cu_x_Sb_2_Cl_12_ microcrystals. The time course of evolution on c) CO and d) CH_4_ of Cs_4_Mn_1‐x_Cu_x_Sb_2_Cl_12_ microcrystals. e) Photocatalytic stability measurement of the Cs_4_Mn_0.7_Cu_0.3_Sb_2_Cl_12_ microcrystals. f) O_2_ yield of Cs_4_MnSb_2_Cl_12_ and Cs_4_Mn_0.7_Cu_0.3_Sb_2_Cl_12_ microcrystals.

The photocatalytic performances of Cs_4_Mn_1‐x_Cu_x_Sb_2_Cl_12_ microcrystals were obtained by continuous irradiation with a 300 W Xe lamp for 3 h. **Figure** **3** (a) shows the CO and CH_4_ yields of Cs_4_Mn_1‐x_Cu_x_Sb_2_Cl_12_ microcrystals after 3 h continuous illumination. With the introduction of Cu, the photocatalytic reduction ability of Cs_4_Mn_1‐x_Cu_x_Sb_2_Cl_12_ microcrystals was improved. Cs_4_Mn_0.7_Cu_0.3_Sb_2_Cl_12_ microcrystals showed the best photocatalytic performance, with CO and CH_4_ yields of 503.86 and 68.35 µmol g^−1^, respectively. The electron consumption was 1007.72 and 546.80 µmol g^−1^, respectively. Since then, with the addition of more Cu, the yield of CO and CH_4_ has gradually decreased. It indicates that the photocatalytic reduction ability of carbon dioxide can be improved by adding appropriate proportion of Cu into Cs_4_MnSb_2_Cl_12_ microcrystals. We compared the CO and CH_4_ yields of Cs_4_Mn_0.7_Cu_0.3_Sb_2_Cl_12_ microcrystals with those of other perovskite photocatalysts previously reported, as shown in Table S3 (Supporting Information). It can be seen that Cs_4_Mn_0.7_Cu_0.3_Sb_2_Cl_12_ has the highest photocatalytic reduction ability and has a good application prospect. In addition, in order to further evaluate the reducing capacity of Cs_4_Mn_1‐x_Cu_x_Sb_2_Cl_12_ microcrystals, we calculated the electron consumption during the reduction process. The formula *R*_electron_ = R(CO)×2+R(CH_4_)×8 is used to obtain the electron consumption of Cs_4_Mn_1‐x_Cu_x_Sb_2_Cl_12_ microcrystals, where R(CO) and R(CH_4_) are the yield of CO and CH_4_, respectively. As shown in Figure 3 (b), Cs_4_Mn_0.7_Cu_0.3_Sb_2_Cl_12_ microcrystals have the highest electron consumption, which is 1544.52 µmol g^−1^, that is, the strongest photocatalytic reduction ability. Figure 3 (c, d) shows the yield curve of CO and CH_4_ with time during the first 3 h of Cs_4_Mn_1‐x_Cu_x_Sb_2_Cl_12_ microcrystals. The Cs_4_Mn_1‐x_Cu_x_Sb_2_Cl_12_ microcrystals yield has a good linear relationship with time, that is, it has a relatively stable photocatalytic reduction ability. Figure 3 (e) shows the change of CO generation rate with time when Cs_4_Mn_0.7_Cu_0.3_Sb_2_Cl_12_ microcrystals are used as photocatalysts under continuous illumination. After 12 h of continuous illumination, the gas formation rate did not decrease significantly, which further proves that Cs_4_Mn_0.7_Cu_0.3_Sb_2_Cl_12_ microcrystal has good stability. In addition, the apparent quantum yield (AQY) was used to evaluate the catalyst efficiency. The AQY of the Cs_4_Mn_0.7_Cu_0.3_Sb_2_Cl_12_ microcrystals was 1.33% after 10 min irradiation with 420 nm monochromatic light. In addition, we also tested the yield of O_2_, as shown in Figure 3 (f).

In order to further test the stability of Cs_4_Mn_0.7_Cu_0.3_Sb_2_Cl_12_ microcrystals after photocatalytic reaction, we took 5.0 mg Cs_4_Mn_0.7_Cu_0.3_Sb_2_Cl_12_ microcrystals and dispersed them on a glass substrate in the same way as described above, and carried out the photocatalytic reaction for 12 h under the same pressure and CO_2_ atmosphere. Then, the microcrystals of Cs_4_Mn_0.7_Cu_0.3_Sb_2_Cl_12_ were characterized by XRD, SEM, and XPS. The XRD results are shown in Figure S16 (a) and are consistent with the original XRD patterns. As shown by SEM in Figure S16 (b,c), (Supporting Information) the morphology of Cs_4_Mn_0.7_Cu_0.3_Sb_2_Cl_12_ microcrystals does not change significantly. The spectrum of XPS is shown in Figure S17 (Supporting Information), and the spectral peaks of each element do not move. These results further verify the good stability of Cs_4_Mn_0.7_Cu_0.3_Sb_2_Cl_12_ microcrystals.

### 2.7 Photoelectric Property

In order to explain the different photocatalytic carbon dioxide reduction abilities of Cs_4_Mn_x_Cu_1‐x_Sb_2_Cl_12_ microcrystals, we further studied their photoelectric properties. We performed time‐resolved photoluminescence (TRPL) tests at an excitation wavelength of 265 nm with a scanning range of 265–799 nm. The time‐resolved photoluminescence spectroscopy are shown in Figure S18 (Supporting Information). The average lifetime of Cs_4_Mn_1‐x_Cu_x_Sb_2_Cl_12_ microcrystals is 14.601, 119.195, 110.490, 114.633, 112.375, and 108.871 µs, respectively (Table S4, Supporting Information). The addition of Cu can greatly improve the average lifetime of Cs_4_MnSb_2_Cl_12_ microcrystals. Longer service life is conducive to more photo‐excited carriers reaching the active site, thus having better carbon dioxide reduction capacity.^[^ ^39^ ^]^

In order to further study the transfer and separation of photoexcited carriers, photocurrent measurements are carried out. As shown in Figure S19 (a) (Supporting Information), the photocurrent intensity of Cs_4_Mn_0.7_Cu_0.3_Sb_2_Cl_12_ is much higher than that of other proportions of microcrystals, which greatly confirms the enhanced carrier mobility and good periodicity in Cs_4_Mn_0.7_Cu_0.3_Sb_2_Cl_12_ microcrystals. In addition, electrochemical impedance spectroscopy (EIS) was further tested (Figure S19 (b)), , Supporting Information) and it can be seen that the charge‐transfer resistance of Cs_4_Mn_1‐x_Cu_x_Sb_2_Cl_12_ microcrystals decreased with the addition of Cu. Although the charge transfer resistance of Cs_4_Mn_0.7_Cu_0.3_Sb_2_Cl_12_ microcrystals is not the lowest, it has the highest photocurrent intensity, thus obtaining the best photocatalytic reduction ability.

### 2.8 Photocatalytic Reduction Mechanism

**Figure 4:**
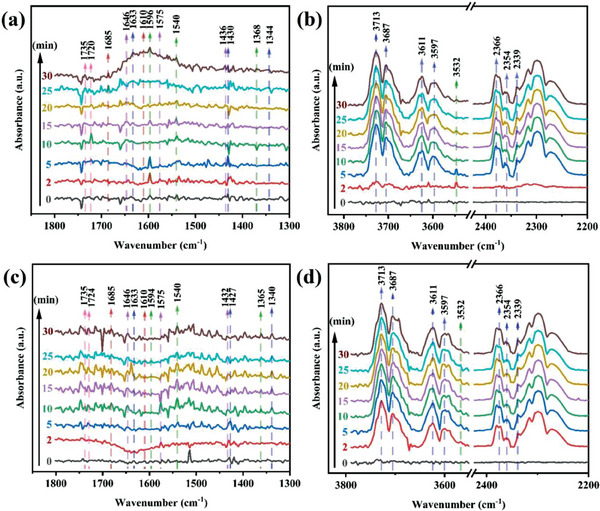
In situ FT‐IR tests for CO_2_ and H_2_O interaction with a, b) Cs_4_Mn_0.7_Cu_0.3_Sb_2_Cl_12_ and c, d) Cs_4_MnSb_2_Cl_12_ in the dark.

In the process of photocatalytic reduction, the photocatalyst plays an important role in the adsorption and activation of CO_2_. In order to investigate the adsorption and activation processes of CO_2_ by Cs_4_MnSb_2_Cl_12_ and Cs_4_Mn_0.7_Cu_0.3_Sb_2_Cl_12_ microcrystals, we performed in situ Fourier transform infrared spectroscopy (FT‐IR). In situ FT‐IR spectra are shown in **Figure** **4** (a, b). Signal peaks of several carbonates and intermediates were detected on the surface of Cs_4_Mn_0.7_Cu_0.3_Sb_2_Cl_12_ microcrystals during the humid CO_2_ adsorption process in dark. The characteristic peaks observed at 1610 and 1685 cm^−1^ are **·**CO^2−^,^[^ ^40^, ^41^ ^]^ which indicates that CO_2_ molecules adsorbed on the surface of Cs_4_Mn_0.7_Cu_0.3_Sb_2_Cl_12_ microcrystals are activated to generate **·**CO^2−^ active intermediates. The peaks at 1368, 1540, and 1596 cm^−1^ were bidentate carbonate species (b‐CO_3_^2−^).^[^ ^39^, ^40^ ^]^ The peaks at 1436, 1575, and 1646 cm^−1^ were monodontic carbonate species (m‐CO_3_^2−^).^[^ ^40^, ^41^, ^42^ ^]^ The peaks at 1720 and 1735 cm^−1^ are chelating‐bridged carbonate species (c‐CO_3_^2−^).^[^ ^40^ ^]^ These carbonates are produced by the formation of carbonic acid on the surface of Cs_4_Mn_0.7_Cu_0.3_Sb_2_Cl_12_ microcrystals and the adsorption of CO_2_. In addition, the characteristic peaks at 1344, 1430 and 1633 cm^−1^ are bicarbonate (HCO_3_^−^),^[^ ^39^, ^43^ ^]^ which is generated by the reaction of CO_2_ and H_2_O adsorbed on the surface. These carbonates and bicarbonates are important intermediates for conversion to CO. The peaks at 2339, 2354 and 2366 cm^−1^ correspond to surface CO_2_ molecules, and the adsorbed gaseous CO_2_ molecules are also detected at 3597, 3611, 3687 and 3713 cm^−1^.^[^ ^39^ ^]^ The peak of OH^−^ was detected at 3532 cm^−1^,^[^ ^44^ ^]^ which was generated from the decomposition of adsorbed H_2_O molecules. In addition, the in situ FT‐IR maps of Cs_4_MnSb_2_Cl_12_ microcrystals are shown in Figure 4 (c, d). The peaks of various carbonates and intermediates are basically consistent with those of Cs_4_Mn_0.7_Cu_0.3_Sb_2_Cl_12_ microcrystals, indicating that the photocatalytic reduction paths of Cs_4_MnSb_2_Cl_12_ and Cs_4_Mn_0.7_Cu_0.3_Sb_2_Cl_12_ microcrystals are the same. In addition, as shown in Figure S20 (Supporting Information), the CO_2_ signal on the surface of Cs_4_Mn_0.7_Cu_0.3_Sb_2_Cl_12_ microcrystals is stronger than that of Cs_4_MnSb_2_Cl_12_ microcrystals, which means that the addition of Cu element can provide more active sites, and also provides strong evidence that Cu element can improve the photocatalytic performance of Cs_4_MnSb_2_Cl_12_ microcrystals.

**Figure 5:**
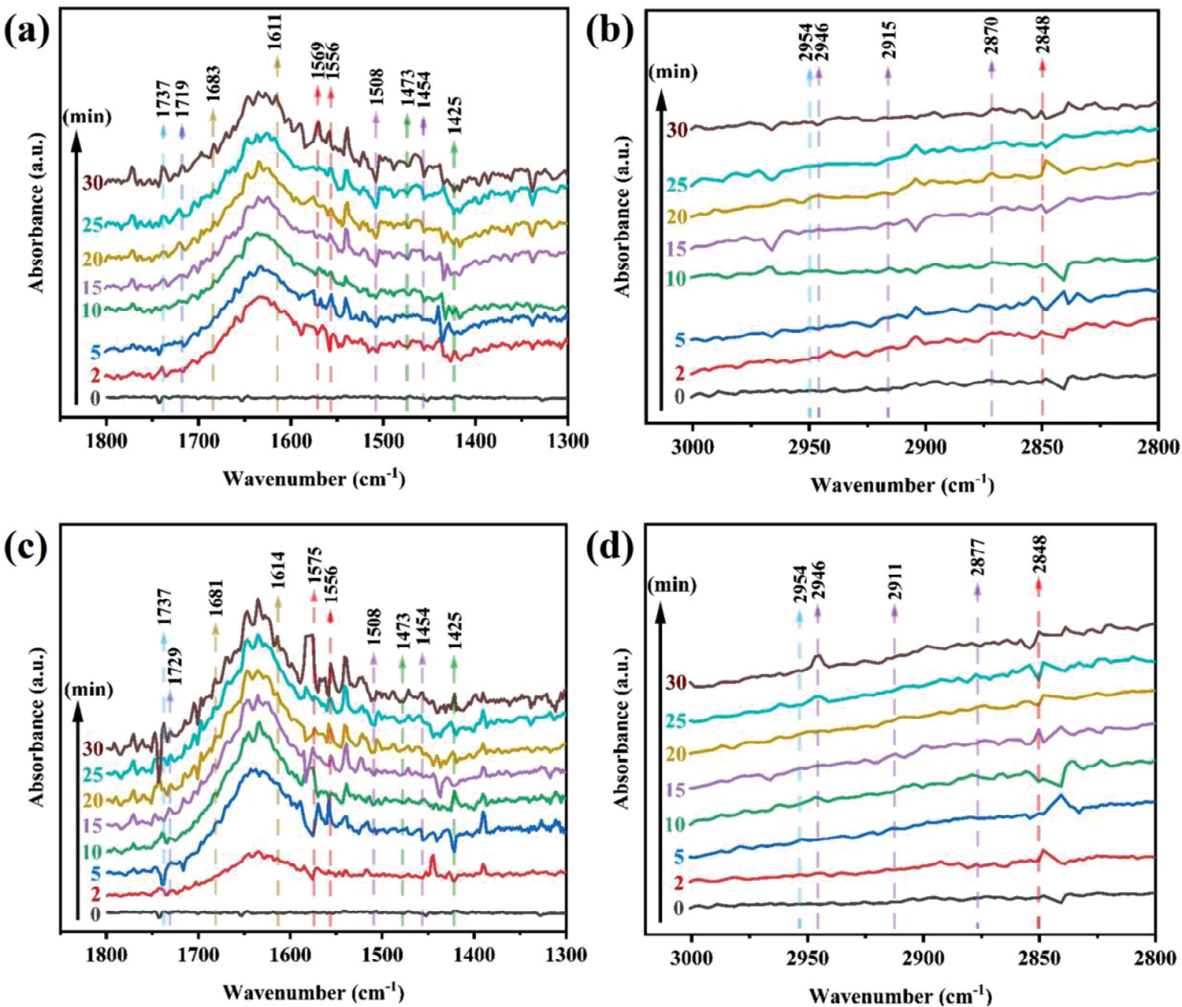
In situ FT‐IR tests of CO_2_ and H_2_O interaction with a, b) Cs_4_Mn_0.7_Cu_0.3_Sb_2_Cl_12_ and c, d) Cs_4_MnSb_2_Cl_12_ under constant 300 W Xe lamp illumination.

We studied the evolution of bonding species on the surface of Cs_4_Mn_0.7_Cu_0.3_Sb_2_Cl_12_ microcrystals under Xe lamp irradiation by in situ FT‐IR. As shown in **Figure** **5** (a, b), the carbonate (CO_3_^2−^) at 1454, 1508 cm^−1^ and the bicarbonate (HCO_3_^−^) at 1425, 1473 cm^−1^ were adsorbed and activated and converted to other substances with increasing light duration.^[^ ^40^, ^41^, ^45^, ^46^ ^]^ After the beginning of illumination, the peaks at 1556 and 1569 cm^−1^, 1719^−1^, 1611 and 1685 cm^−1^ were **·**COOH, CO**·,** and **·**CO_2_^−^,^[^ ^41^, ^47^, ^48^ ^]^ respectively, and continued to rise with the increase of illumination time, which confirmed the generation of CO gas. In addition, a number of intermediates were produced during the reaction, such as CH_3_O**·** (2848 cm^−1^), CH_2_ **·** (2870, 2915, 2946 cm^−1^) and CH_3_ **·** (2954 cm^−1^),^[^ ^49^, ^50^, ^51^, ^52^, ^53^ ^]^ which confirmed the presence of CH_4_ gas. We also found a c‐CO_3_^2−^ absorption peak at 1737 cm^−1^, the intensity of which increased with the duration of the light. This indicates that in the process of photocatalytic reduction, intermediate products accumulate on the surface of Cs_4_Mn_0.7_Cu_0.3_Sb_2_Cl_12_, occupying a part of the catalytic active site, which also confirms the reason why the linear relationship between the yield curve of CO and CH_4_ is not particularly good. In addition, in situ FT‐IR tests were also conducted on Cs_4_MnSb_2_Cl_12_ microcrystals, and the results were obtained as shown in Figure 5 (c, d). The peak positions of various substances were basically the same as those of Cs_4_Mn_0.7_Cu_0.3_Sb_2_Cl_12_ microcrystals.

**Figure 6:**
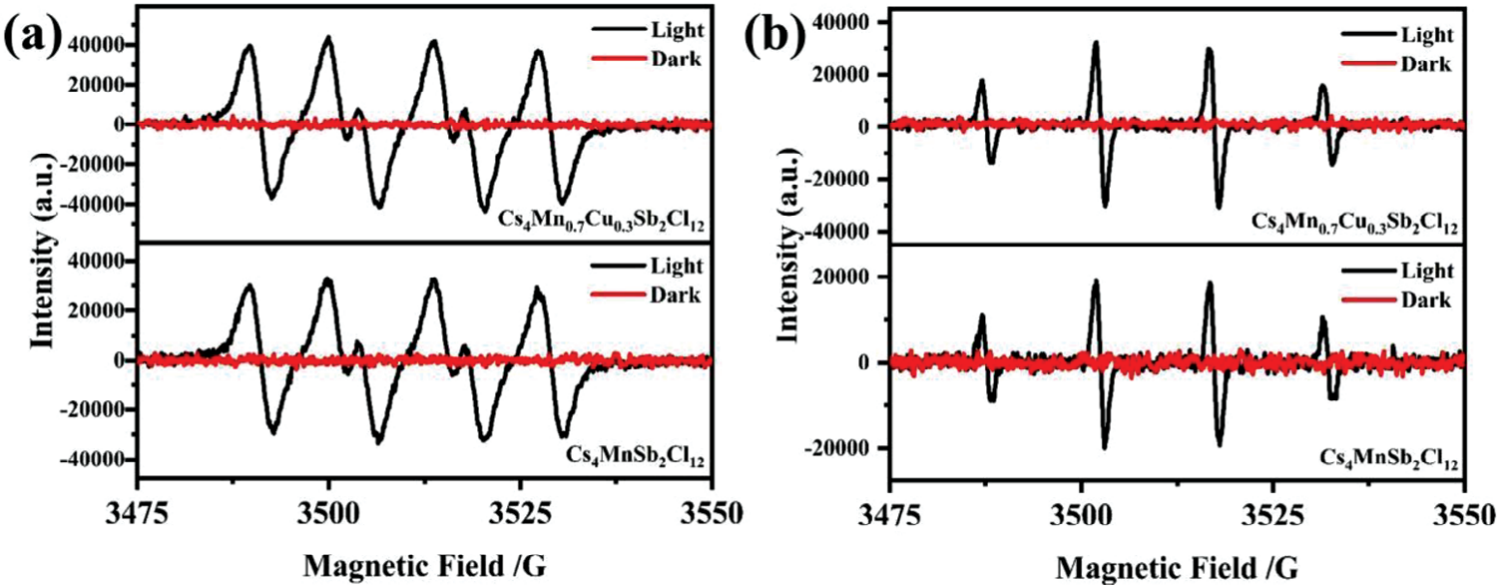
ESR profiles for a) DMPO‐**·**O_2_ **^−^** and b) DMPO‐**·**OH for Cs_4_MnSb_2_Cl_12_ and Cs_4_Mn_0.7_Cu_0.3_Sb_2_Cl_12_ microcrystals.

In addition, we performed an electron spin resonance (ESR) test using 5,5‐dimethyl‐pyrroline‐N‐oxide (DMPO) as a trapping agent to search for oxidation intermediates during photocatalysis. **Figure** **6** (a, b) shows the changes of DMPO‐**·**O_2_ **^−^** and DMPO‐**·**OH before and after illumination. No obvious characteristic peak was detected in the dark condition, indicating that DMPO‐**·**O_2_ **^−^** and DMPO‐**·**OH species were not produced on the surface of Cs_4_MnSb_2_Cl_12_ and Cs_4_Mn_0.7_Cu_0.3_Sb_2_Cl_12_ microcrystals without light irradiation. After 10 min of irradiation with a 300 W Xe lamp, a clear characteristic peak of DMPO‐**·**O_2_ **^−^** and DMPO‐**·**OH were detected, indicating that superoxide and hydroxyl radicals were generated after illumination, leading to the excitation of e**^−^** and h**^+^**.^[^ ^54^ ^]^ We also found that the intensity of DMPO‐**·**O_2_ **^−^** and DMPO‐**·**OH characteristic peaks of Cs_4_Mn_0.7_Cu_0.3_Sb_2_Cl_12_ microcrystals is higher than that of Cs_4_MnSb_2_Cl_12_ microcrystals, which is consistent with the ability of photocatalytic activity, further proving that the addition of Cu element can obtain more photoexcited carriers, and thus have higher photocatalytic performance.

**Figure 7:**
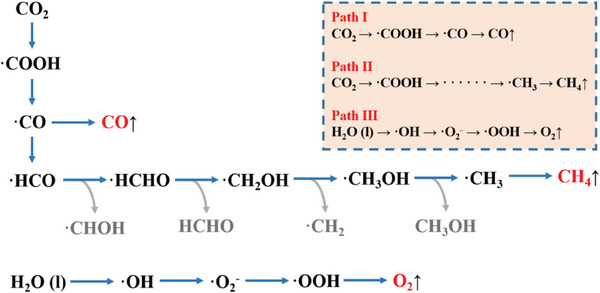
Reaction mechanism of photocatalytic production of CO, CH_4,_ and O_2_ by Cs_4_Mn_1‐x_Cu_x_Sb_2_Cl_12_ microcrystals.

Through in situ FT‐IR and ESR spectra, the specific process of photocatalytic reaction of Cs_4_Mn_1‐x_Cu_x_Sb_2_Cl_12_ microcrystals can be obtained, as shown in **Figure** **7**.

## 3 Conclusion

In this work, we report the synthesis and characterization of a series of <111> inorganic double perovskites by the formula Cs_4_Mn_1‐x_Cu_x_Sb_2_Cl_12_ (x = 0, 0.1, 0.2, 0.3, 0.4, and 0.5). The photocatalytic reduction performance of Cs_4_Mn_1‐x_Cu_x_Sb_2_Cl_12_ microcrystals was studied for the first time. The photocatalytic reduction performance of Cs_4_Mn_0.7_Cu_0.3_Sb_2_Cl_12_ microcrystals was the best. After 3 h illumination, the yields of CO and CH_4_ were 503.86 and 68.35 µmol g^−1^, which exceeded many previously reported photocatalysts. After 12 h of photocatalytic reaction, high temperature treatment and 365 nm UV irradiation, Cs_4_Mn_0.7_Cu_0.3_Sb_2_Cl_12_ microcrystals can still maintain good stability. In addition, through a series of tests, we can know that the addition of Cu element can make Cs_4_MnSb_2_Cl_12_ microcrystals capture more photoexcited carriers and have higher characteristic peak intensity, so as to obtain better photocatalytic performance Compared with the original Cs_4_MnSb_2_Cl_12_, the Cu‐doped Cs_4_MnSb_2_Cl_12_ has a smaller bandgap, higher photoexcited carrier separation capability, and higher photocurrent intensity, which further verifies its excellent photocatalytic performance. This study shows the potential of Cs_4_Mn_1‐x_Cu_x_Sb_2_Cl_12_ microcrystals as clean, stable, and efficient photocatalysts.

## 4 Experimental Section

### Materials

CsCl (99.9%) and Sb_2_O_3_ (99.99%) were purchased from Aladdin, MnCl_2_ (99%) were purchased from HEOWNS, CuCl_2_ (98%) was purchased from Macklin, HCl (37 wt.% in water) and Ethanol (>99.7%) were purchased from Chongqing Wansheng East Sichuan Chemical Co. LTD. All the chemicals were used without further purification.

### Synthesis of Cs4Mn1‐xCuxSb2Cl12 Microcrystals

First, according to the stoichiometric ratio, 1 mmol Sb_2_O_3_ (291.5 mg) and 1 mmol MnCl_2_ (125.8 mg) were added to 10 mL of hydrochloric acid, heated and stirred at 60 °C until the powder was completely dissolved, and then 4 mmol CsCl (673.4 mg) was added to continue stirring. After being completely dissolved, the product was washed with anhydrous ethanol, the mixed solution was centrifuged at 3000 rpm for 5 min, the supernatant was removed, and the Cs_4_MnSb_2_Cl_12_ microcrystals were obtained by vacuum drying at 60 °C for 12 h. By the same method, the molar ratio of MnCl_2_ and CuCl_2_ was adjusted to obtain Cs_4_Mn_1‐x_Cu_x_Sb_2_Cl_12_ microcrystals (x = 0, 0.1, 0.2, 0.3, 0.4, and 0.5). The specific ratio is shown in Table S1. (Supporting Information)

### Characterization

X‐ray diffraction (XRD) spectra of Cs_4_Mn_1‐x_Cu_x_Sb_2_Cl_12_ (x = 0, 0.1, 0.2, 0.3, 0.4, and 0.5) microcrystals were determined by Cu Kα diffraction (MADZU, Japan). The apparent morphology of Cs_4_Mn_1‐x_Cu_x_Sb_2_Cl_12_ microcrystals was characterized by scanning electron microscopy (SEM, TM4000Plus II). The transmission electron microscopy (TEM) and high‐resolution transmission electron microscopy (HRTEM) were performed on ZEISS LIBRA 200FE. Element mappings were tested by energy dispersive X‐ray spectroscopy (EDX) on the same instrument. Inductively coupled plasma‐atomic emission spectrometry (ICP‐AES) was tested on an Agilent 730. The UV‐visible absorption spectra were determined by the scan UV–vis spectrophotometer (UV‐2100) (Shimadzu, Japan) with a range of 300 to 800 nm.The chemical states and valence bands of Cs_4_Mn_1‐x_Cu_x_Sb_2_Cl_12_ microcrystals were characterized by X‐ray photoelectron spectroscopy (XPS, ESRCALAB250Xi, Thermo Fisher Scientific). The binding energy referred to the C 1s peak at the binding energy of 284.80 eV. Thermogravimetric analysis (TGA) studies were performed in AR environments from room temperature to 1000 °C using a TGA/DSC analyzer (METTLER TOLEDO, and Switzerland). Time‐resolved photoluminescence (TRPL) spectra were measured using a photoluminescence spectrometer (Cary Eclipse G9800A, Agilent Technologies).

### Stability Test

The stability of Cs_4_Mn_1‐x_Cu_x_Sb_2_Cl_12_ microcrystals was tested in the following environment. In the first case, 50.0 mg sample is placed under the high temperature at 100 °C for 100 h. In the second case, 50.0 mg sample is exposed to light at a wavelength of 365 nm for 100 h. Then, the treated samples were analyzed by XRD, SEM, and XPS.

### Photocatalytic CO_2_ Reduction

The photocatalytic carbon dioxide reduction was studied by Labsolar‐6A system (Perfect Light Co., China). The system consists of a fully enclosed quartz reactor, a 300 W Xe lamp, and a gas chromatography (GC). 300 W Xe lamp as a photocatalytic light source. 2.0 mg Cs_4_Mn_1‐x_Cu_x_Sb_2_Cl_12_ (x = 0, 0.1, 0.2, 0.3, 0.4, and 0.5) microcrystals were weighed and added to 1 mL anhydrous ethanol. After ultrasonic dispersion for 30 min, the mixture was coated on a glass substrate (2.0 × 2.0 cm) and heated at 100 °C for 1 h to remove the excess anhydrous ethanol. The treated glass piece was placed in a 100 mL sealed Pyrex glass bottle, degassed repeatedly to remove air, and then, CO_2_ gas with purity of 99.999% was filled to keep the reaction pressure in the glass bottle at 85–90 kPa, and 10 µL of deionized water was injected. After collecting two sets of blank data, illumination was started. At the end of each hour of the reaction, the collected gas was evaluated by gas chromatography.

### Apparent Quantum Yield

To measure the apparent quantum yield (AQY) of the photocatalytic reaction system, a LED lamp with 420 nm monochromatic light (Shenzhen LAMPLIC Science Co. China) was used as the light source. Except for changing the light source, all of them are carried out under the photocatalytic reaction conditions mentioned above. The illuminated area was controlled as a circle with a diameter of 1.5 cm, and the incident monochromatic illumination intensity was 50 mW cm^−2^. The AQY was calculated from Equation (1).

(1) $$\mathrm{AQY}\;=\;\frac{\mathrm{Number}\;\mathrm{of}\;\mathrm{reacted}\;\mathrm{electrons}}{\mathrm{Number}\;\mathrm{of}\;\mathrm{incident}\;\mathrm{photons}}\times 100\%$$

(2) $$\begin{array}{ccc} & = & \;\frac{\mathrm{Number}\;\mathrm{of}\;\mathrm{CO}\;\mathrm{molecules}\times 2+\mathrm{Number}\;\mathrm{of}\;\mathrm{CH}4\;\mathrm{molecules}\times 8}{\mathrm{Number}\;\mathrm{of}\;\mathrm{incident}\;\mathrm{photons}}\\ & & \times 100\% \end{array}$$

### Photoelectrochemical Characterizations

Indium tin oxide (ITO) substrates (1.5 × 1.5 cm) were cleaned sequentially with deionized water, alcohol and acetone for 10 min each. 2.0 mg Cs_4_Mn_1‐x_Cu_x_Sb_2_Cl_12_ (x = 0, 0.1, 0.2, 0.3, 0.4, and 0.5) microcrystals were added into 1 mL anhydrous ethanol, and after ultrasonic treatment for 30 min, they were dripped on ITO substrate, and then heated and dried at 60 °C to remove excess supernatant. The photoelectrochemical experiments were carried out on CHI760e in a three‐electrode configuration, with the assembled photoelectrode (Cs_4_Mn_1‐x_Cu_x_Sb_2_Cl_12_ microcrystals spin‐coated on ITO) as the working electrode, the Pt net as the reverse electrode, and the Ag/AgCl electrode as the reference electrode. A mixture of 0.1 m tetrafluorobutyl ammonium phosphate (TBAPF_6_) and ethyl acetate was used as the electrolyte. A Xe lamp was used to simulate sunlight, and the bias potential was set to 0.4 V, and the change of photoinduced current density with time (*i–t* curve) was recorded. The electrochemical reactions in this process are^[^ ^28^ ^]^

(3) $${\mathrm{TBA}}^{+}+O_{2}+e^{-}\to {\mathrm{TBAO}}_{2}$$

(4) $${\mathrm{PF}}_{6}^{-}+H_{2}O-4e^{-}\to {\mathrm{HPF}}_{6}+O_{2}$$

In addition, the EIS Nyquist curve of Cs_4_Mn_1‐x_Cu_x_Sb_2_Cl_12_ microcrystals was tested by setting the bias potential to 0.25 V, the amplitude to 0.005 V, the low frequency to 82.5 Hz, and the high frequency to 1e+5 Hz.

### In Situ FT‐IR Investigation on Photocatalytic CO_2_ Reduction

We took the in situ FT‐IR test on the BRUKER TENSOR 27 Fourier Transform infrared spectrometer, which is equipped with a reactor and liquid nitrogen cooled MCT detectors. Prior to the test, the samples were cleaned with Ar (50 mL min^−1^) at 120 °C for 1 h to remove impurities on the surface of the samples. The background spectra were collected after the reactor temperature was lowered to room temperature. Then Ar (25 mL min^−1^), CO_2_ (5 mL min^−1^) and trace H_2_O vapor were introduced into the reactor to record the change of FT‐IR spectrum. The background spectrum was collected again after the adsorption equilibrium (≈30 min) was reached. The reaction process was then studied by using 300 W Xe lamp irradiation and recording FT‐IR spectra.

### Electron Spin Resonance (ESR) Test

The test was conducted on a Brooker‐A300 model of equipment. First, 1.0 mg mL^−1^ sample solution was prepared, and 200 µL of the solution was added into 200 µL DMPO solution after homogenization by ultrasound. After mixing evenly, the solution was loaded with capillary samples for testing, and tested once in the dark condition and under 300 W Xe lamp irradiation for 10 min.

## Conflict of Interest

The authors declare no conflict of interest.

## Supporting information

Supporting Information

## Data Availability

The data that support the findings of this study are available from the corresponding author upon reasonable request.
