# Supplementary material for: Copper Modulated Lead‐Free Cs4MnSb2Cl12 Double Perovskite Microcrystals for Photocatalytic Reduction of CO2
Source: Adv Sci (Weinh). 2023 Dec 9;11(6):2307543. doi: 10.1002/advs.202307543 (PMC10853743; doi:10.1002/advs.202307543)
Supplement: Supplementary file 1 — Supporting Information [file ADVS-11-2307543-s001.pdf]

ADVANCED SCIENCE
Open Access

Supporting Information

for *Adv. Sci.*, DOI 10.1002/advs.202307543

Copper Modulated Lead-Free $Cs_4MnSb_2Cl_{12}$ Double Perovskite Microcrystals for Photocatalytic Reduction of CO_2

Bo Gao, Changqing Tian, Linfeng Guo, Jinchen Zhou, Zixian Wang, Chengfan Fu, Hongmei Ran, Wei Chen, Qiang Huang, Daofu Wu, Xiaosheng Tang* and Zhongtao Luo**

Supporting Information

Copper Modulated Lead-Free $Cs_4MnSb_2Cl_{12}$ Double Perovskite Microcrystals for Photocatalytic Reduction of CO_2

Bo Gao[a], *Changqing Tian*[b], *Linfeng Guo*[b], *Jinchen Zhou*[b], *Zixian Wang*[b], *Chengfan Fu*[b], *Hongmei Ran*[b], *Wei Chen*[b], *Qiang Huang*[b], *Daofu Wu*[c,]*, *Xiaosheng Tang*[a,b,d,]*, *Zhongtao Luo*[a,]*

[a] School of Materials Science and Engineering, Zhengzhou University, Zhengzhou 450001, China

[b] College of Optoelectronic Engineering, Chongqing University of Posts and Telecommunications, Chongqing 400065, China

[c] State Key Laboratory of Catalysis, Dalian Institute of Chemical Physics, Chinese Academy of Sciences, Dalian 116023, China

[d] Key Laboratory of Optoelectronic Technology & Systems (Ministry of Education), College of Optoelectronic Engineering, Chongqing University, Chongqing 400044, China

*** Corresponding author**

E-mail: wudaofu@dicp.ac.cn (D. Wu), xstang@cqu.edu.cn (X. Tang), luozhongtao@126.com (Z. Luo).

Table S1. Mass of copper and manganese precursors added during the synthesis of $Cs_4Mn_{1-x}Cu_xSb_2Cl_{12}$.

X	$MnCl_2$ Mass (mg)	$CuCl_2$ Mass (mg)
**0.1**	**113.2**	**13.4**
**0.2**	**100.6**	**26.9**
**0.3**	**88.1**	**40.3**
**0.4**	**75.5**	**53.8**
**0.5**	**62.9**	**67.2**

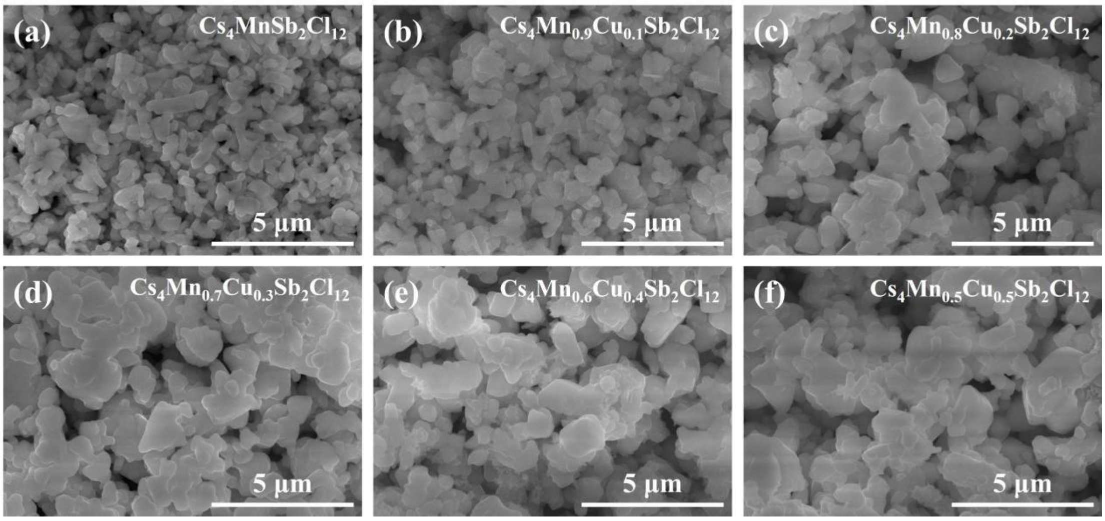

Figure S1. (a~f) SEM images of $Cs_4Mn_{1-x}Cu_xSb_2Cl_{12}$ microcrystals.

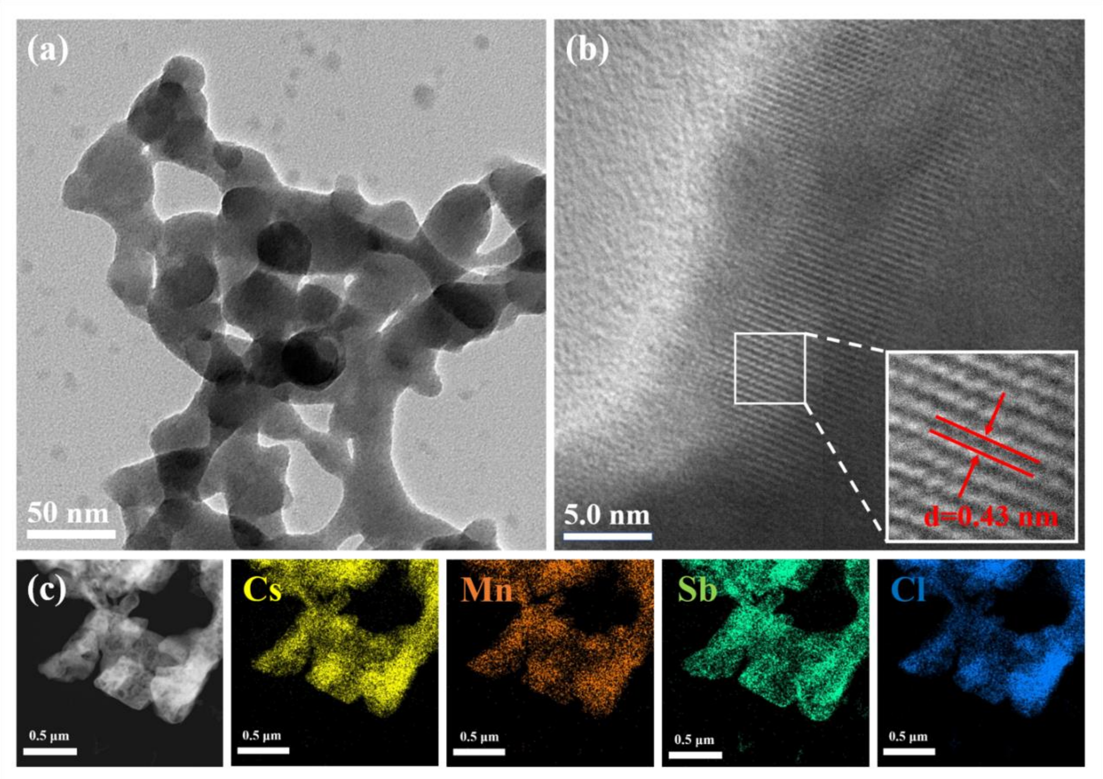

Figure S2. (a,b) TEM and HRTEM images of $Cs_4MnSb_2Cl_{12}$ microcrystals. (c) EDX mapping results of $Cs_4MnSb_2Cl_{12}$ microcrystals.

Table S2. Atomic percentage of elements present in the synthesized catalysts.

**Measurement**	**Atomic percentage**	
	**Mn**	**Cu**
$\mathbf{Cs_4MnSb_2Cl_{12}}$	**4.643**	**/**
$\mathbf{Cs_4Mn_{0.9}Cu_{0.1}Sb_2Cl_{12}}$	**3.989**	**0.488**
$\mathbf{Cs_4Mn_{0.8}Cu_{0.2}Sb_2Cl_{12}}$	**3.409**	**0.991**
$\mathbf{Cs_4Mn_{0.7}Cu_{0.3}Sb_2Cl_{12}}$	**3.468**	**1.539**
$\mathbf{Cs_4Mn_{0.6}Cu_{0.4}Sb_2Cl_{12}}$	**2.916**	**2.033**
$\mathbf{Cs_4Mn_{0.5}Cu_{0.5}Sb_2Cl_{12}}$	**2.456**	**2.654**

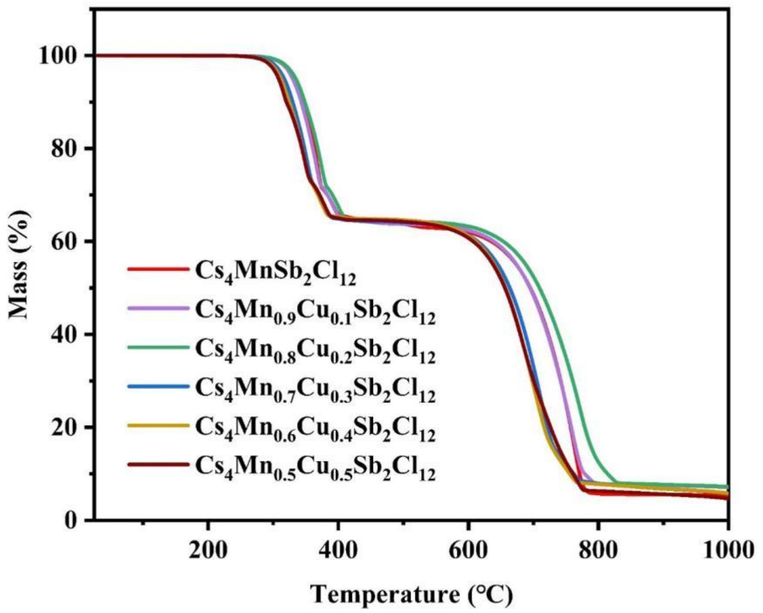

Figure S3. TGA curves of the $Cs_4Mn_{1-x}Cu_xSb_2Cl_{12}$ microcrystals.

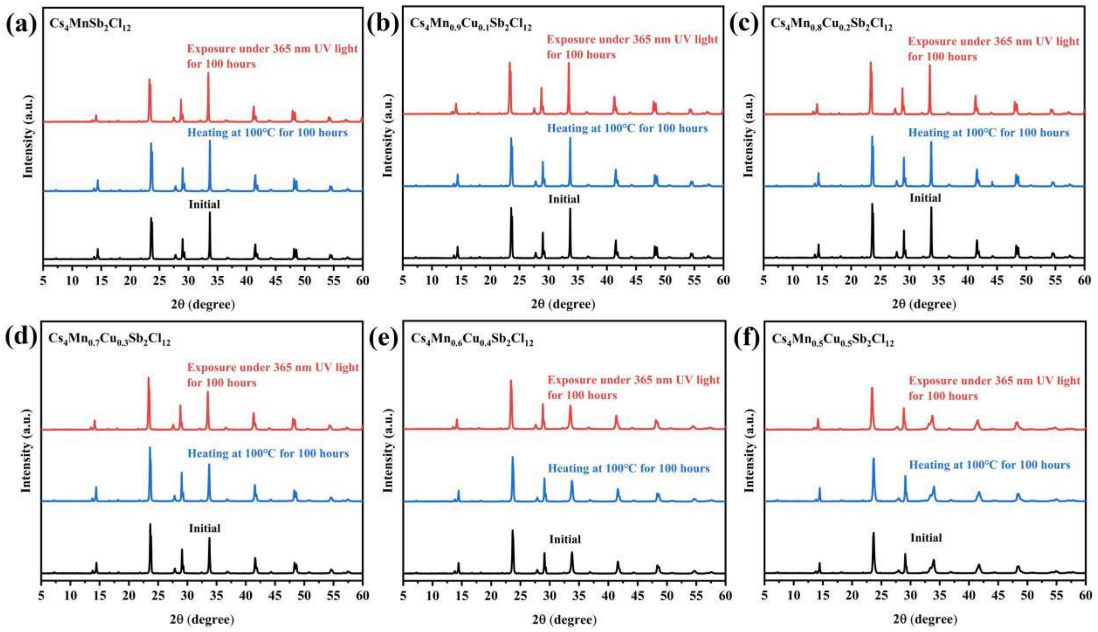

Figure S4. (a~f) XRD patterns of $Cs_4Mn_{1-x}Cu_xSb_2Cl_{12}$ microcrystals before and after high temperature treatment and irradiation by 365 nm UV lamp.

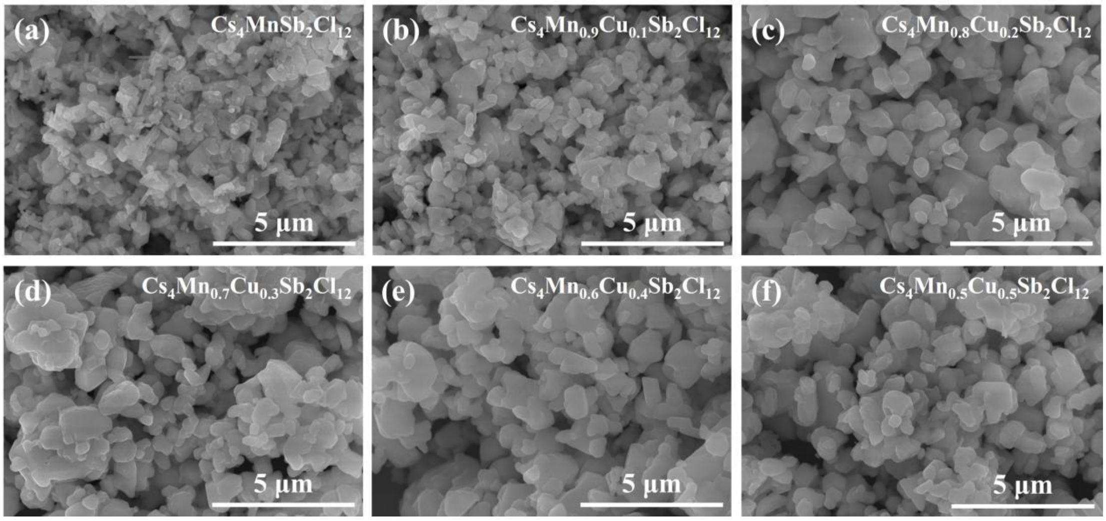

Figure S5. (a~f) SEM images of $Cs_4Mn_{1-x}Cu_xSb_2Cl_{12}$ microcrystals after 100 hours at 100 ℃.

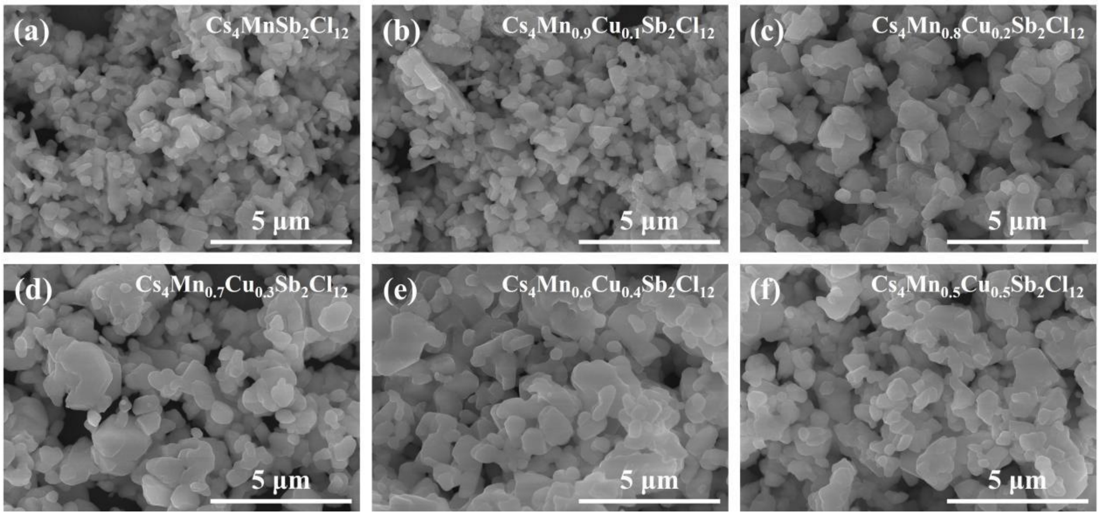

Figure S6. (a~f) SEM images of $Cs_4Mn_{1-x}Cu_xSb_2Cl_{12}$ microcrystals after 100 hours irradiation by 365 nm UV lamp.

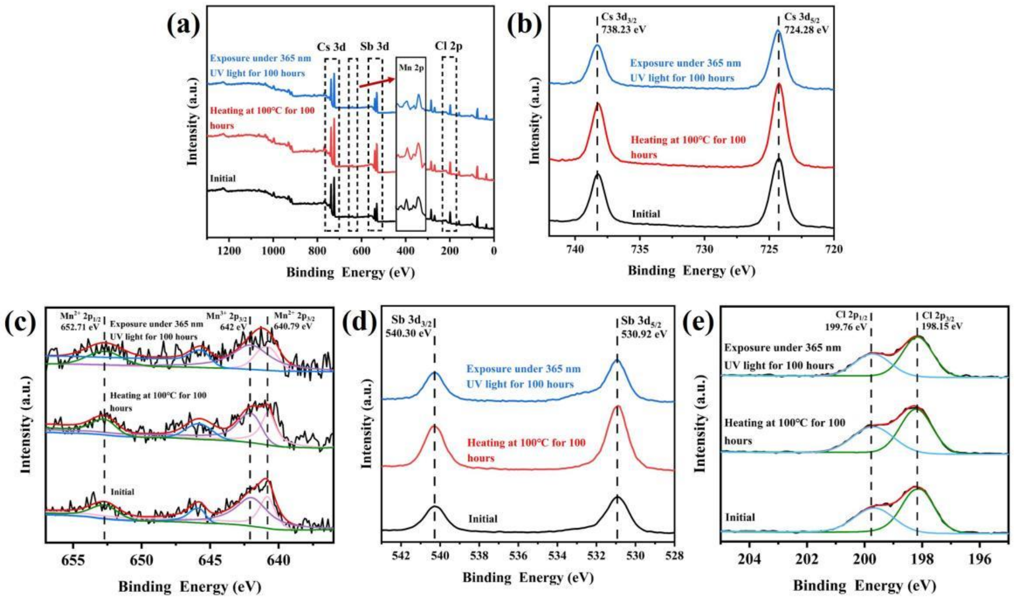

Figure S7. (a) XPS spectra of $Cs_4MnSb_2Cl_{12}$ microcrystals before and after high temperature treatment and irradiation by 365 nm UV lamp, (b) Cs 3d, (c) Mn 2p, (d) Sb 3d, (e) Cl 2p.

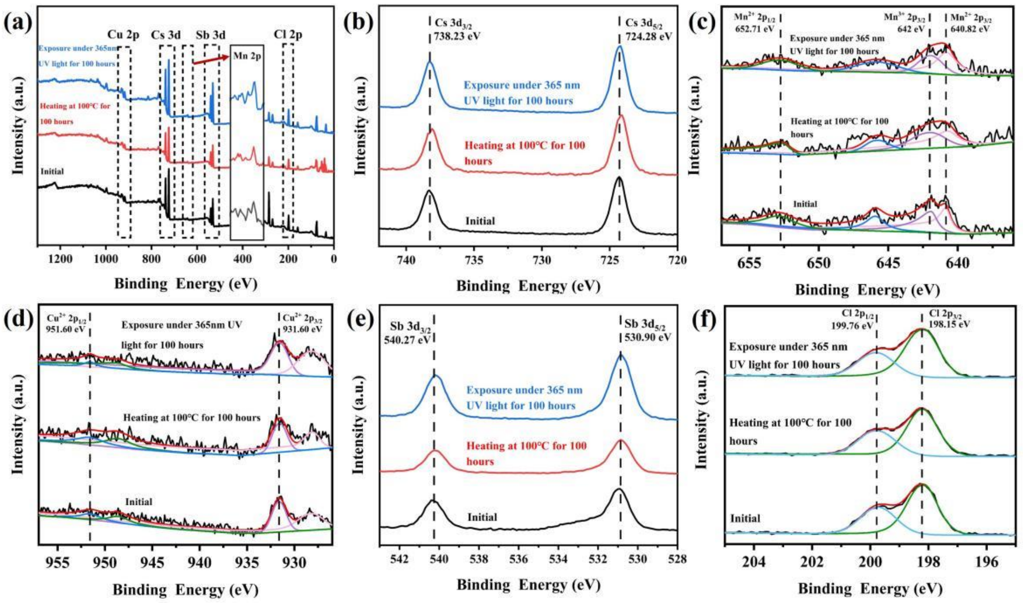

Figure S8. (a) XPS spectra of $Cs_4Mn_{0.9}Cu_{0.1}Sb_2Cl_{12}$ microcrystals before and after high temperature treatment and irradiation by 365 nm UV lamp, (b) Cs 3d, (c) Mn 2p, (d) Cu 2p, (e) Sb 3d, (f) Cl 2p.

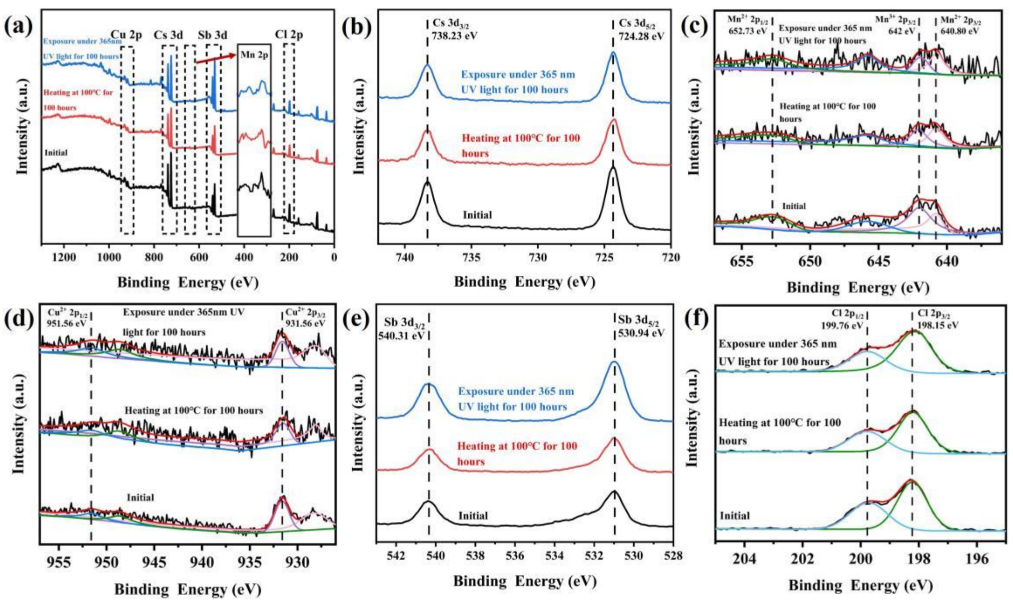

Figure S9. (a) XPS spectra of $Cs_4Mn_{0.8}Cu_{0.2}Sb_2Cl_{12}$ microcrystals before and after high temperature treatment and irradiation by 365 nm UV lamp, (b) Cs 3d, (c) Mn 2p, (d) Cu 2p, (e) Sb 3d, (f) Cl 2p.

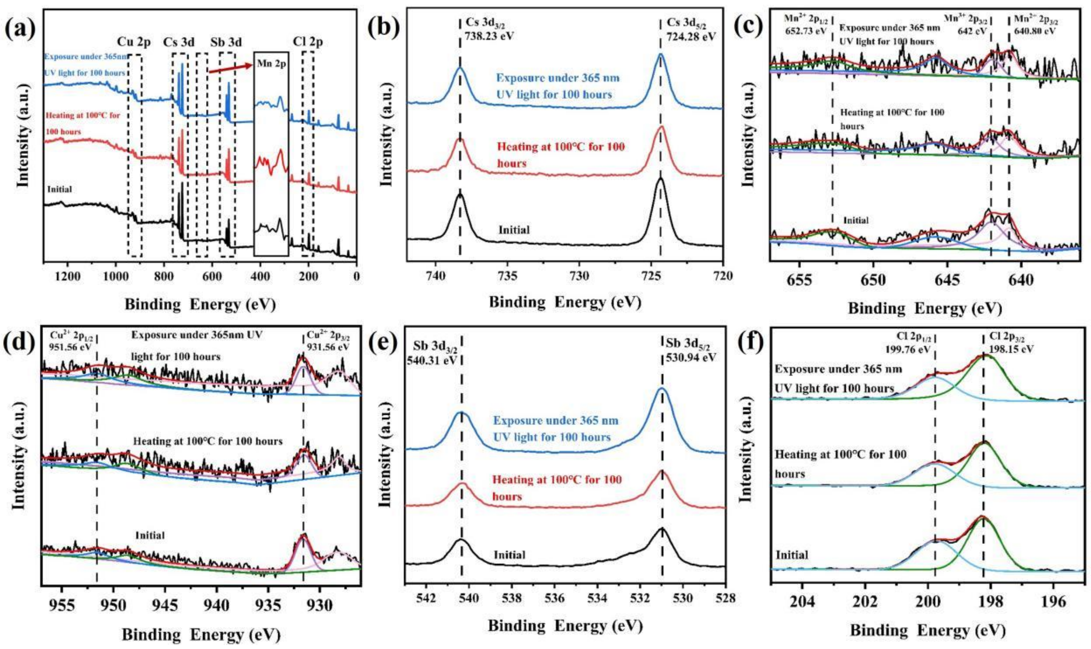

Figure S10. (a) XPS spectra of $Cs_4Mn_{0.7}Cu_{0.3}Sb_2Cl_{12}$ microcrystals before and after high temperature treatment and irradiation by 365 nm UV lamp, (b) Cs 3d, (c) Mn 2p, (d) Cu 2p, (e) Sb 3d, (f) Cl 2p.

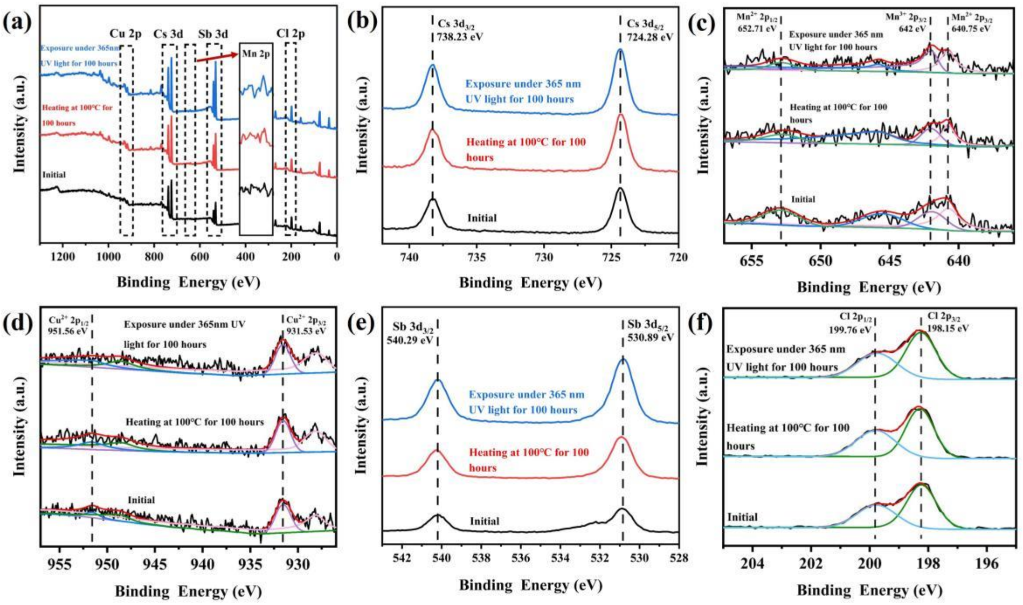

Figure S11. (a) XPS spectra of $Cs_4Mn_{0.6}Cu_{0.4}Sb_2Cl_{12}$ microcrystals before and after high temperature treatment and irradiation by 365 nm UV lamp, (b) Cs 3d, (c) Mn 2p, (d) Cu 2p, (e) Sb 3d, (f) Cl 2p.

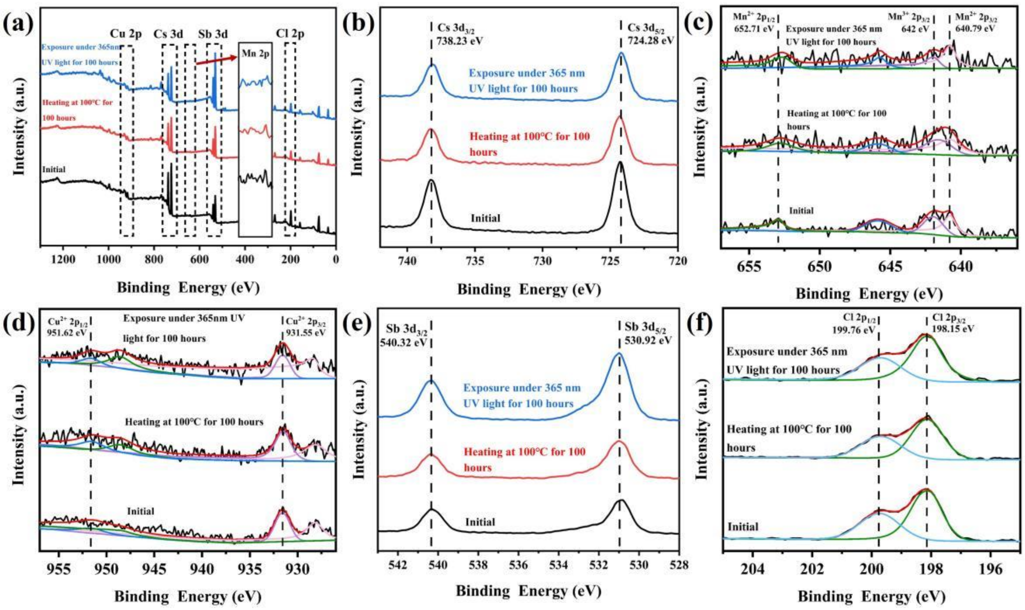

Figure S12. (a) XPS spectra of $Cs_4Mn_{0.5}Cu_{0.5}Sb_2Cl_{12}$ microcrystals before and after high temperature treatment and irradiation by 365 nm UV lamp, (b) Cs 3d, (c) Mn 2p, (d) Cu 2p, (e) Sb 3d, (f) Cl 2p.

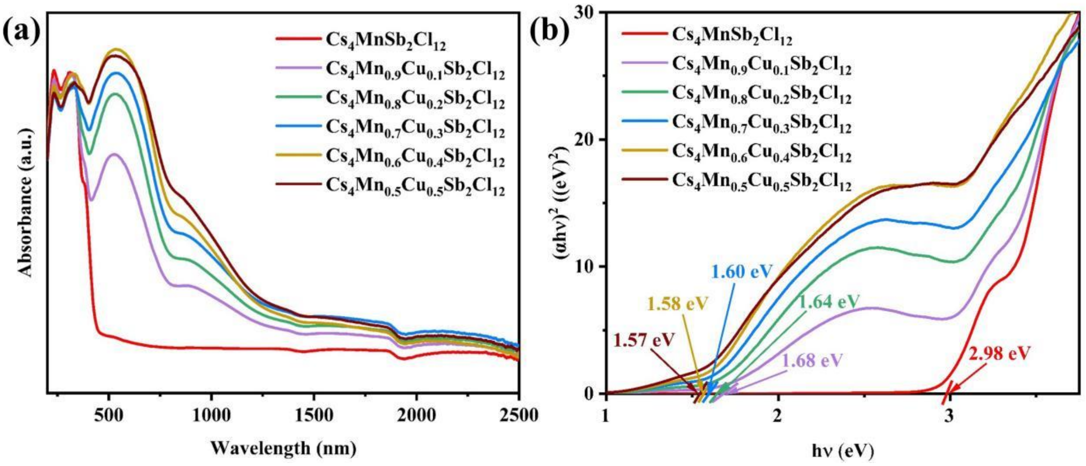

Figure S13. (a) UV-vis absorption spectra of the $Cs_4Mn_{1-x}Cu_xSb_2Cl_{12}$. (b) $(\alpha h\nu)^2$ versus hν curves of the $Cs_4Mn_{1-x}Cu_xSb_2Cl_{12}$ microcrystals.

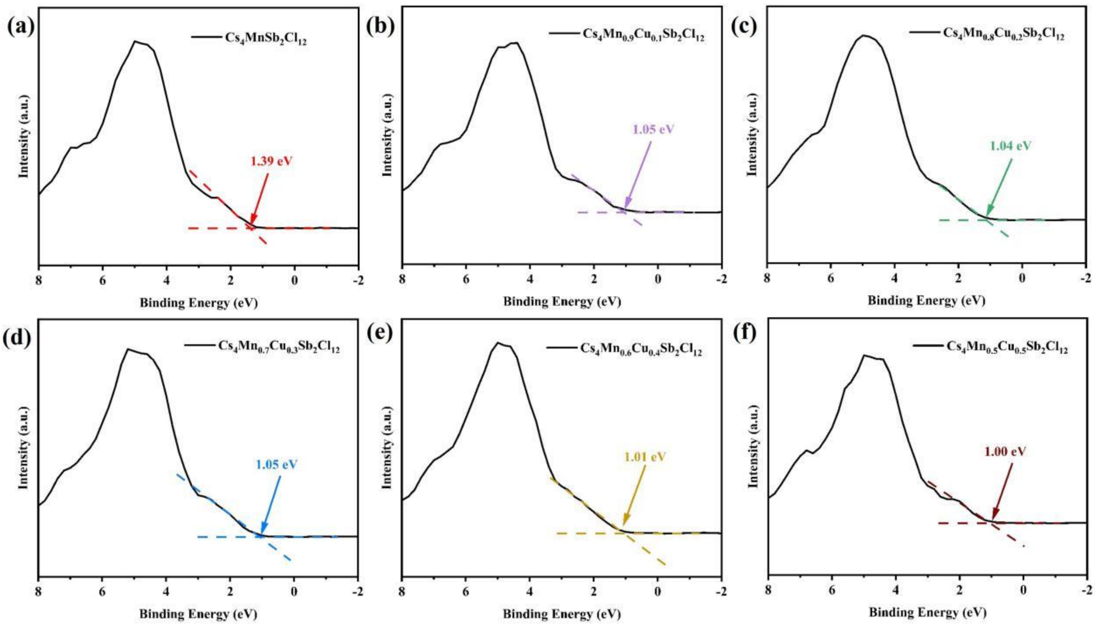

Figure S14. (a~f) VB XPS spectra of $Cs_4Mn_{1-x}Cu_xSb_2Cl_{12}$ microcrystals.

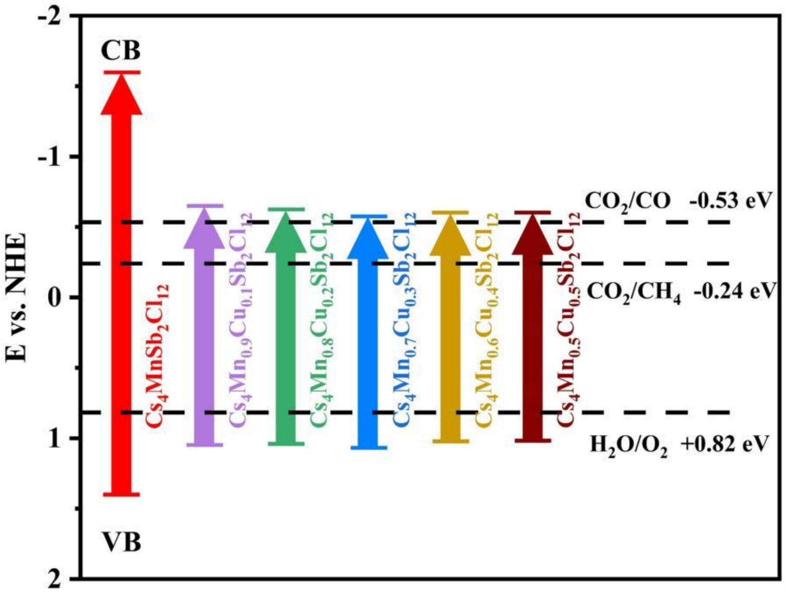

Figure S15. Schematic illustration of the energy band structures of $Cs_4Mn_{1-x}Cu_xSb_2Cl_{12}$ microcrystals.

Table S3. Comparison of the photocatalytic CO_2 reduction activity and CO selectivity of various perovskites-based photocatalysts.

Catalysts	Products ($\mu molg^{-1}h^{-1}$)	Selectivity/%	Cocatalyst	Light	References
**$Cs_4Mn_{0.7}Cu_{0.3}Sb_2Cl_{12}$**	**CO=167.95 CH_4=22.78**	**88.06**	**/**	**Xe, 300W**	**This work**
Cs_2TeCl_6	CO=94.8 CH_4=16.3	85.3	/	Xe, 300W	[1]
$Cs_4CuSb_2Cl_{12}$ QDs	CO=77.67 CH_4=24.67	75.9	/	Xe, 300W	[2]
CABB@MCM-48	CO=120.0 CH_4=17.0	87.6	/	λ ≥ 420 nm	[3]
Cu/$CsPbBr_3$-Cs_4PbBr_6 NCs	CO=58.5 CH_4=53.7	52.1	/	AM 1.5G, 100 mW cm^{-2}	[4]
Mn:$CsPbCl_3$ NCs	CO=37.0	/	/	Xe, 300W	[5]
$CsPbBr_{2.4}I_{0.6}$	CO=43.9	/	/	Xe 300W,	[6]
ZnSe−$CsSnCl_3$	CO=57.0 CH_4=1.79	97.0	/	Xe 300W,	[7]
Cu-Bpy-COF	CO=13.0 CH_4=45.9	22.1	COF	λ ≥ 420 nm	[8]

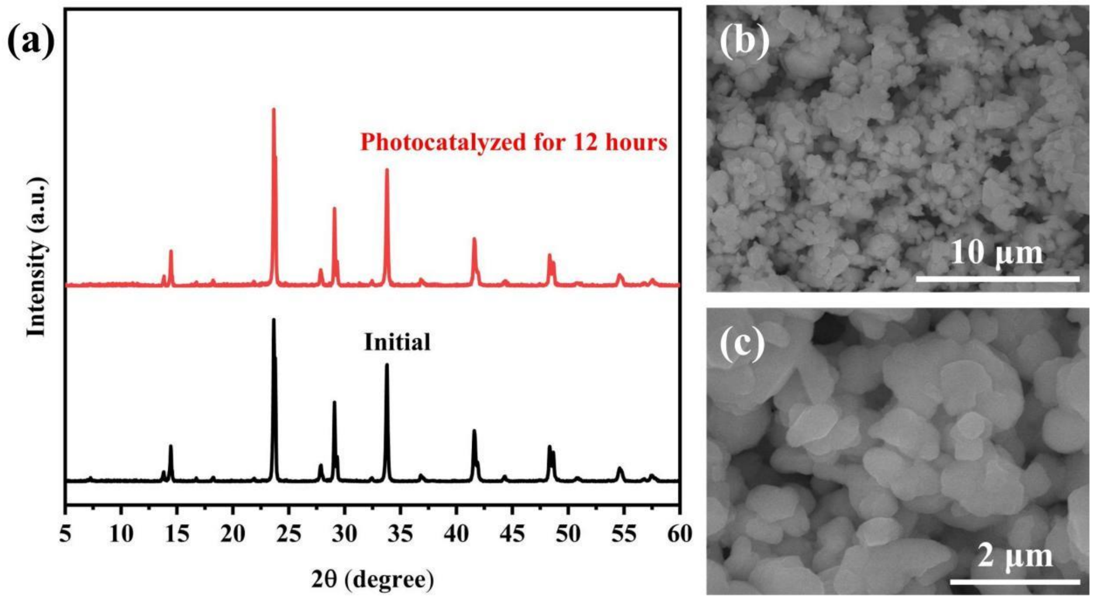

Figure S16. (a) XRD patterns of $Cs_4Mn_{0.7}Cu_{0.3}Sb_2Cl_{12}$ microcrystals before and after photocatalytic reduction reaction. (b,c) SEM images of $Cs_4Mn_{0.7}Cu_{0.3}Sb_2Cl_{12}$ microcrystals after photocatalytic reduction reaction.

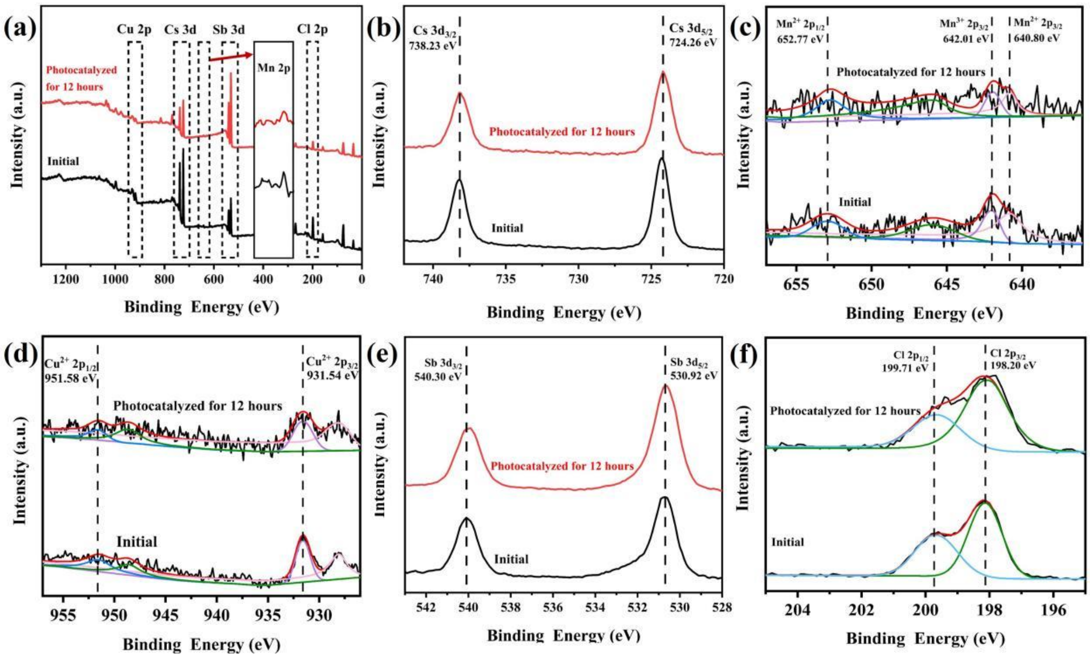

Figure S17. (a) XPS spectra of the $Cs_4Mn_{0.7}Cu_{0.3}Sb_2Cl_{12}$ microcrystals before and after photocatalytic reduction reaction, (b) Cs 3d, (c) Mn 2p, (d) Cu 2p, (e) Sb 3d, (f) Cl 2p.

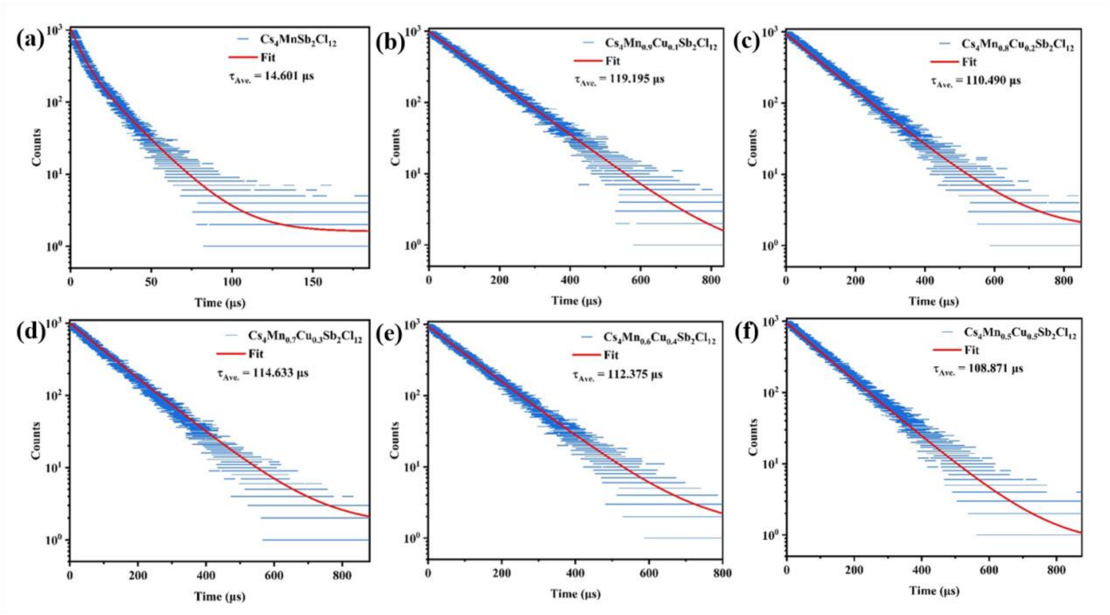

Figure S18. Time-resolved photoluminescence (TRPL) spectroscopy of $Cs_4Mn_{1-x}Cu_xSb_2Cl_{12}$ microcrystals.

Table S4. TRPL decay parameters of the $Cs_4Mn_{1-x}Cu_xSb_2Cl_{12}$ microcrystals.

**X**	**τ_1 (μs)**	**A_1**	**τ_2 (μs)**	**A_2**	**$\tau_{Ave.}$ (μs)**
**0**	**0.646**	**6.012**	**0.397**	**19.020**	**14.601**
**0.1**	**0.537**	**117.796**	**0.460**	**120.788**	**119.195**
**0.2**	**0.467**	**98.457**	**0.467**	**120.336**	**110.490**
**0.3**	**0.499**	**102.149**	**0.499**	**124.848**	**114.633**
**0.4**	**0.472**	**100.136**	**0.472**	**122.389**	**112.375**
**0.5**	**0.474**	**97.014**	**0.474**	**118.572**	**108.871**

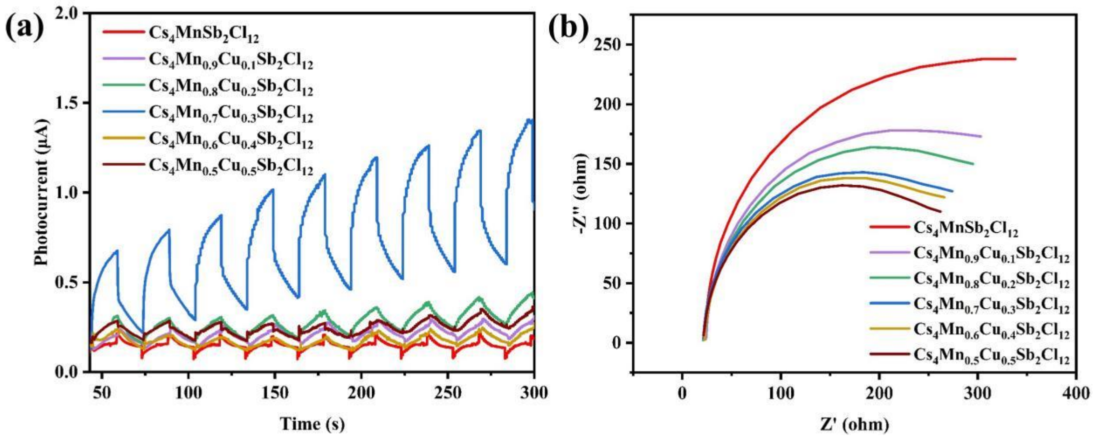

Figure S19. (a) Transient photocurrent responses at 0.1 V versus Ag/AgCl electrode and (b) EIS Nyquist plots at a bias of 0.25 V Ag/AgCl under irradiation in the ethyl acetate solution containing 0.1 M tetrabutylammonium hexaflfluorophosphate (TBAPF6).

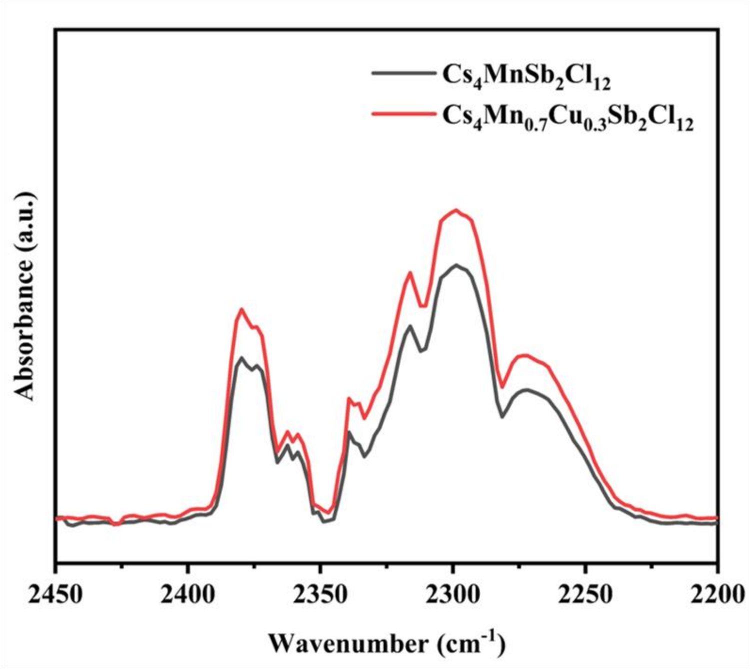

Figure S20. Sorption equilibrium spectra of $Cs_4MnSb_2Cl_{12}$ and $Cs_4Mn_{0.7}Cu_{0.3}Sb_2Cl_{12}$ microcrystals after CO_2 and H_2O adsorption in the dark for 10 minutes.

References

[1] C. Q. Tian, Q. Huang, D. F. Wu, J. A. Lai, F. Qi, N. Zhang, W. X. Zhang, X. S. Tang, *Mater. Today Energy* **2022**, 28, 101067.

[2] D. F. Wu, C. Q. Tian J. C. Zhou, Y. Y. Huang, J. A. Lai, B. Gao, P. Heng, Q. Huang, X. S. Tang, *Carbon Neutralization* **2022**, 1, 298.

[3] Z. J. Zhang, D. B. Li, Z. L. Dong, Y. Jiang, X. Li, Y. Q. Chu, J. Y. Xu, *Solar RRL* **2023**, 7, 2300038.

[4] L. J. Li, Z. H. Zhang, *Chem. Eng. J.* **2022**, 434, 134811.

[5] J. Wang, L. Xiong, Y. Bai, Z. J. Chen, Q. Zheng, Y. Y. Shi, C. Zhang, G. C. Jiang, Z. Q. Li, *Solar RRL* **2022**, 6, 2200294.

[6] G. Q. Wang, F. J. Zhang, Y. R. Wang, J. Ma, *ChemNanoMat* **2022**, 8, e202200230.

[7] N. Y. Li, X. J. Chen, J. Wang, X. M. Liang, L. T. Ma, X. L. Jing, D. L. Chen, Z. Q. Li, *ACS Nano* **2022**, 16, 3332.

[8] Y. Z. Zhang, L. L. Cao, G. Y. Bai, X. W. Lan, *Small* **2023**, 19, e2300035.
